# Area dependent behavior of bathocuproine (BCP) as cathode interfacial layers in organic photovoltaic cells

**DOI:** 10.1038/s41598-018-30826-7

**Published:** 2018-08-22

**Authors:** Bhushan R. Patil, Mehrad Ahmadpour, Golnaz Sherafatipour, Talha Qamar, Antón F. Fernández, Karin Zojer, Horst-Günter Rubahn, Morten Madsen

**Affiliations:** 1 SDU nanoSYD, Mads Clausen Institute, University of Southern Denmark, Alsion 2, 6400 Sønderborg, Denmark; 2 Institute of Solid State Physics and NAWI Graz, Graz University of Technology, Petersgasse 16, 8010 Graz, Austria

## Abstract

Standard and inverted configuration small molecule OPV cells incorporating bathocuproine (BCP) as electron transport and exciton blocking layer is investigated, demonstrating that 2 mm^2^ standard and inverted cells display a maximum performance for BCP thicknesses of 10 nm and 1.5 nm, respectively. The reason for the different optimum BCP thicknesses for the two device configurations is the BCP-metal complex formed between the Ag electrode and the BCP layer in the standard configuration OPV devices. Interestingly, at optimum BCP thicknesses, the inverted OPV cells outperform the standard devices. Upon up-scaling of the device area of the cells from 2 mm^2^ to 10 and 100 mm^2^, device failure becomes prominent for the inverted OPV cells, due to aggregation of the evaporated BCP layer on the ITO surface. This demonstrates that although BCP can be adopted for efficient ETL in inverted configuration OPV devices on small scale, it is not suitable for device up-scaling due to severely decreasing device yields. In this work, a possible solution where an ultrathin layer of C_70_ is evaporated between the ITO and BCP layer is proposed. It is demonstrated that the proposed solution holds a strong potential to minimize the device failures of the BCP based inverted OPV cells to a significant extent, while maintaining good device performances.

## Introduction

Organic photovoltaics (OPVs), being eco-friendly and easy-to-produce, are considered to be a prominent sustainable energy source for the future. To date, in the development of highly efficient OPVs, fullerene and their derivatives are dominantly used as electron acceptor layers^1–3^. However, although fullerenes demonstrate excellent electron mobilities, efficient charge extraction in OPV devices still requires an Electron Transport and Exciton Blocking Layer (ETL and EBL, respectively) with well-matched energy levels, that is integrated between the electron acceptor layer and the metal cathode, in order to minimize any interface losses^4–9^. A plain interface between the metal cathode and the fullerene acceptor layer gives rise to multiple losses, be that due to insufficient exciton blocking or recombination effects. Particularly severe non-radiative recombination losses occur when a metal is evaporated on top of fullerene layers, because the metal atoms penetrate into the fullerene film^10^. In order to enhance the electron collection efficiently in OPVs and to reduce potential exciton losses at the fullerene-metal interface, it is thus common to sandwich proper ETL and EBL in between the fullerene acceptor and metal cathode^6–9,11,12^. The combined ETL and EBL may also act as a protective layer for the acceptor from damage during the metal deposition^6,10,11,13^.

**Figure 1:**
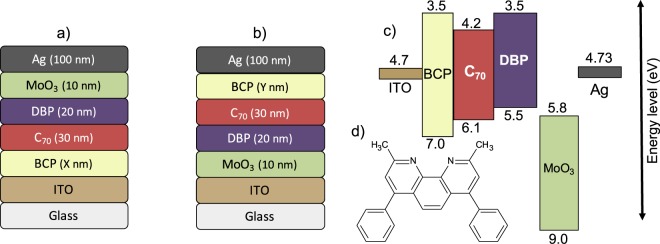
OPV device layer stack with (**a**) inverted and (**b**) standard configuration; (**c**) energy level diagram of OPVs with inverted configuration and (**d**) the molecular structure of BCP.

2,9-Dimethyl-4,7-diphenyl-1,10-phenanthroline (Bathocuproine, BCP) is an organic material that is being widely used as ETL in OPV devices to improve electron extraction in the cells^14^. The molecular structure of BCP shown in Fig. 1d. BCP thin films can be evaporated at very low temperatures and possess a high optical transparency due to BCP’s large bandgap^13^. BCP has a deep lying Highest Occupied Molecular Orbital (HOMO) level at 7.0 eV, which combined with a Lowest Unoccupied Molecular Orbital (LUMO) at 3.5 eV, provides excellent exciton blocking properties for the most commonly used material systems in OPV^12,15,16^. Furthermore, Vogel *et al*. reported that a thin layer of BCP inserted in phthalocyanine (Pc) and C_60_ based standard configuration OPV cells improves the Power Conversion Efficiency (PCE) of the devices remarkably^16^. This increase in the PCE is due to a reduction of non-radiative recombination effects at the interface of C_60_ and Al; without BCP, the non-radiative recombination region extends into the C_60_ layer due to Al penetration^16^.

Despite its high bandgap, BCP facilitates efficient electron transport in standard configuration OPV devices^17–20^. This transport efficiency is ascribed to the presence of metal-BCP complexes that form when the metal, here Ag, is evaporated on top of the BCP layer. This complex formation requires a strong interaction between the BCP molecule and the metal atom^21,22^. The LUMO level of the formed Ag-BCP complex aligns with the LUMO level of the fullerene acceptor layers, which facilitates the efficient electron transport seen in the OPV devices^22^. Inverted OPVs lack this favorable alignment of BCP and fullerene LUMO levels, because metal-BCP complexes are not formed as the BCP layer is deposited directly on top of the bottom electrode in inverted devices. The less favorable LUMO level alignment hampers efficient electron extraction in BCP based inverted OPV devices^21^. The detailed role of the BCP layer for electron transport in inverted OPV devices is to date, however, not fully investigated.

In this work, we assess the impact of BCP as electron transport and exciton blocking layer on the performance of standard and inverted planar heterojunction OPV devices consisting of Tetraphenyldibenzoperiflanthene (DBP) as donor and Fullerene (C_70_) as acceptor molecules. DBP and C_70_ solar cells have been extensively studied in the past, reaching power conversion efficiencies of up to 6.4% for single, mixed heterojunction solar cells^23,24^, whereas the bilayer OPVs with DBP and C_70_ usually exhibit a device performance of around 3 to 3.5%^4,25^. We are particularly interested in the change of the OPV device performance when scaling up the device area, in particular when BCP films of different thicknesses are incorporated. We demonstrate that an up-scaling of the device area from 2 mm^2^ to 10 and 100 mm^2^ leads to distinctively different device yields for the standard and inverted configuration. The yields of standard configuration OPV devices were not affected by up-scaling to 10 and 100 mm^2^. However, with up-scaling from 2 mm^2^ to 10 and 100 mm^2^ a strongly increasing amount of inverted OPV devices failed due to shunting. We attribute the shunting to extended BCP aggregates that form upon evaporation on Indium-Tin-Oxide (ITO). Device failure due to BCP aggregation strongly reduces the overall OPV device yield. To improve the yield of the inverted devices upon up-scaling, we propose a simple method for reducing the aggregation of BCP on ITO by sandwiching an ultra-thin layer of C_70_ in between the ITO and BCP layers. The proposed method improves the overall device yield as well as the performance of the fabricated 100 mm^2^ inverted OPV devices.

## Results and Discussion

**Figure 2:**
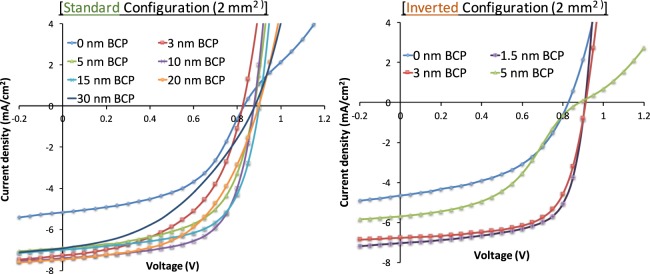
JV characteristics of 2 mm^2^ standard and inverted OPVs with various BCP thicknesses as electron transport layer.

The JV characteristics of the standard and inverted OPV devices of the 2 mm^2^ cell area with various thicknesses of the BCP interfacial cathode layer are shown in Fig. 2. The associated performance parameters are listed in Tables 1 and 2, respectively. The energy level diagram of the inverted OPV devices with the BCP layer is shown in Fig. 1c.

**Table 1:** The performance parameters of 2 mm^2^ standard configuration OPV devices with various thicknesses of the BCP layer.

BCP thickness (nm)	Voc (mV)	J_sc_ (mA/cm^2^)	FF (%)	PCE (%)
0	817 ± 27	4.95 ± 0.33	49 ± 04	1.99 ± 0.31
3	826 ± 25	6.81 ± 0.07	42 ± 02	2.20 ± 0.13
5	880 ± 10	6.93 ± 0.18	59 ± 02	3.61 ± 0.19
**10**	**871** ± **13**	**7**.**32** ± **0**.**17**	**63** ± **01**	**4**.**06** ± **0**.**10**
15	899 ± 03	7.09 ± 0.26	62 ± 01	4.00 ± 0.17
20	886 ± 35	7.15 ± 0.17	49 ± 04	3.11 ± 0.40
30	872 ± 11	6.49 ± 0.23	41 ± 07	2.33 ± 0.46

**Table 2:** The performance parameters of 2 mm^2^ inverted configuration OPV devices with various thicknesses of BCP layer.

BCP thickness (nm)	V_oc_ (mV)	J_sc_ (mA/cm^2^)	FF (%)	PCE (%)
0	851 ± 10	4.11 ± 0.19	52 ± 03	2.27 ± 0.08
**1**.**5**	**910** ± **03**	**7**.**04** ± **0**.**07**	**70** ± **02**	**4**.**48** ± **0**.**13**
3	915 ± 02	6.94 ± 0.17	64 ± 02	4.08 ± 0.07
5	888 ± 06	5.29 ± 0.32	45 ± 03	2.09 ± 0.13

For the JV characteristics of the standard OPV devices with various BCP thicknesses (Fig. 2a), the devices with 10 nm of BCP (violet curve) show the highest J_SC_, FF and PCE. Standard cells without BCP show ‘S-shaped’ JV characteristics (blue curve). The S-shape is likely caused by recombination losses at Ag clusters that penetrated into the C_70_ layer, reminiscent to the reported case of Al cathodes^16^. For the standard configuration cells, increasing the BCP thickness up to 15 nm almost doubles the PCE of the devices compared to the devices without BCP; the PCE enhancement is mostly due to increased J_SC_ and FF (Table 1). Increasing the thickness of the BCP layer above 15 nm in the standard configuration cells significantly decreases the device PCE by a factor of 1.7, along with reduced FF and J_sc_. The occurrence of optimum performance values around 10–15 nm has also been reported elsewhere^6,15,17,26^. The reduction in performance beyond 15 nm BCP thickness is presumably caused by the competition between two coexisting sub-regions within the BCP layer, namely a pristine and a metal-permeated BCP region, i.e. a region containing the aforementioned BCP-Ag complex^26^. The pristine BCP region poses a barrier for electron transport, while the metal-permeated BCP region enables an efficient electron extraction in the devices. When the thickness of the BCP layer increases above 15 nm, the transport occurs in the dominant pristine BCP region, translating into poor FF and PCE values. Thus, an optimized thickness of the BCP layer is crucial for obtaining high device performances.

BCP layers are also crucial for inverted device structures but support the function of the OPV device in a different fashion. In the BCP-free inverted device, J_SC_ as low as 4.1 mA/cm^2^ (Table 2) and low FF (blue curve in Fig. 2b) are encountered, since exciton blocking is absent and the carrier extraction at the interface between C_70_ and ITO is inefficient. Inserting the BCP layer as ETL in between ITO and C_70_ in the inverted OPV devices establishes an improved cathode contact and significantly enhances the short circuit current J_SC_ to more than 5.3 mA/cm^2^ (Table 2). Since metal-BCP complexes cannot be formed, transport has to be established across a pristine BCP region. The decrease in the device performance with increasing BCP thickness, clearly reflected in the JV characteristics shown in Fig. 2b, appears to predominantly arise due to the resistance of the unaffected BCP layer (Table 2).

**Figure 3:**
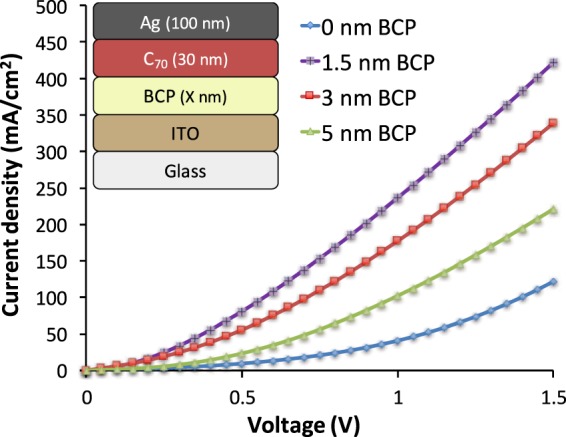
The JV characteristics of the inverted Electron-Only-Devices (EODs) with 0, 1.5, 3 and 5 nm BCP as ETL; EOD device layer stack is shown in inset.

To corroborate the hypothesis of a detrimental impact of the BCP layer resistance on the current transport, we inspect the JV characteristics of inverted electron-only devices (EODs), shown in Fig. 3. When operating an EOD, electrons were injected through Ag electrode and collected at the ITO electrode. The JV-characteristics of the EODs follow the same trends as in the OPVs. At the low BCP thickness of 1.5 nm, the EOD clearly shows an improved electron extraction (violet curve) compared to reference devices without BCP (blue) curve, in line with the high device performance of inverted configuration cells with 1.5 nm BCP thickness. An increase in the thickness of BCP above 1.5 nm deteriorates the electron current due to a lessened conductivity through the BCP layer. Hence, inverted cells require much thinner optimal BCP layer thicknesses compared to the standard cells.

Remarkably, the inverted cells with BCP films thinner than 5 nm (red curve in Fig. 2b) outperform the optimized standard configuration cells (BCP thickness 10 nm), even though BCP-Ag complexes, that would favor electron transport, are absent. Also, the BCP-containing inverted cells are superior to inverted cells using ZnO rather than BCP as ETL in an otherwise identical structure^4^.

**Figure 4:**
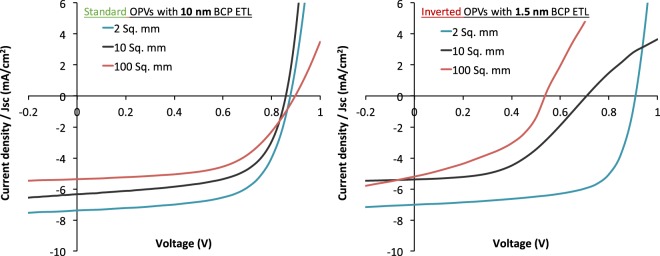
JV characteristics of standard OPVs with BCP ETL of 10 nm and inverted OPVs with BCP ETL of 1.5 nm with the cell areas of 2, 10 and 100 mm^2^.

To probe whether the OPV devices with their configuration-specifically optimized BCP layers keep their performance also in larger cells, OPV devices with 10 nm and 1.5 nm thick BCP layers for standard and inverted configurations, respectively, were scaled up from cell areas of 2 to 10 and 100 mm^2^. The JV characteristics of the OPV devices with cells areas of 2, 10 and 100 mm^2^ are shown in Fig. 4. The related performance parameters including the device yield are listed in Tables 3 and 4. Overall, the PCE of the devices decreases while scaling up the cell area. This decrease in PCE is expected, because the increase in the area of the ITO electrode area causes a larger resistance in ITO and, hence, a reduction in J_SC_ and FF (Tables 3 and 4)^1,27^.

**Table 3:** The performance parameters of 2, 10 and 100 mm^2^ standard OPVs with BCP ETL of 10 nm.

OPV cell area (mm^2^)	V_OC_ (mV)	J_SC_ (mA/cm^2^)	FF (%)	PCE (%)	Device yield (%)
2	871 ± 13	7.32 ± 0.17	63 ± 01	4.06 ± 0.10	≈100%
10	857 ± 21	6.43 ± 0.07	63 ± 01	3.50 ± 0.13	≈100%
100	897 ± 27	5.29 ± 0.15	57 ± 02	2.70 ± 0.13	≈100%

**Table 4:** The performance parameters of 2, 10 and 100 mm^2^ inverted OPVs with BCP ETL of 1.5 nm.

OPV cell area (mm^2^)	V_OC_ (mV)	J_SC_ (mA/cm^2^)	FF (%)	PCE (%)	Device yield (%)
2	910 ± 03	7.04 ± 0.07	70 ± 02	4.48 ± 0.13	>90%
10	618 ± 86	5.63 ± 0.41	45 ± 03	1.58 ± 0.24	<40%
100	537 ± 99	5.05 ± 0.21	38 ± 08	1.14 ± 0.11	<10%

Importantly, the overall device yield of the standard configuration OPV devices was close to 100% independent of the cell area, i.e., almost all fabricated devices worked successfully. In contrast, the inverted OPV cells with BCP as interlayer showed a remarkably reduced device yield upon up-scaling. As seen from the performance parameters (Table 4) and JV characteristics (Fig. 4), the optimum performance for the inverted OPV cells with small active area (2 mm^2^) was achieved with 1.5 nm BCP as ETL. The overall device yield for these devices exceeded 90%, i.e., 9 out of 10 fabricated OPV devices worked. When increasing the active area to 10 mm^2^, the device yield dropped to below 40%, and upon further increasing the OPV cell area to 100 mm^2^, the device yield dropped to less than 10%. The majority of the failed OPV devices showed strong leakage currents in their JV characteristics. The less than 10% OPV devices that worked demonstrated a strong decrease in the V_OC_ and FF values and, hence, a strongly reduced PCE.

**Figure 5:**
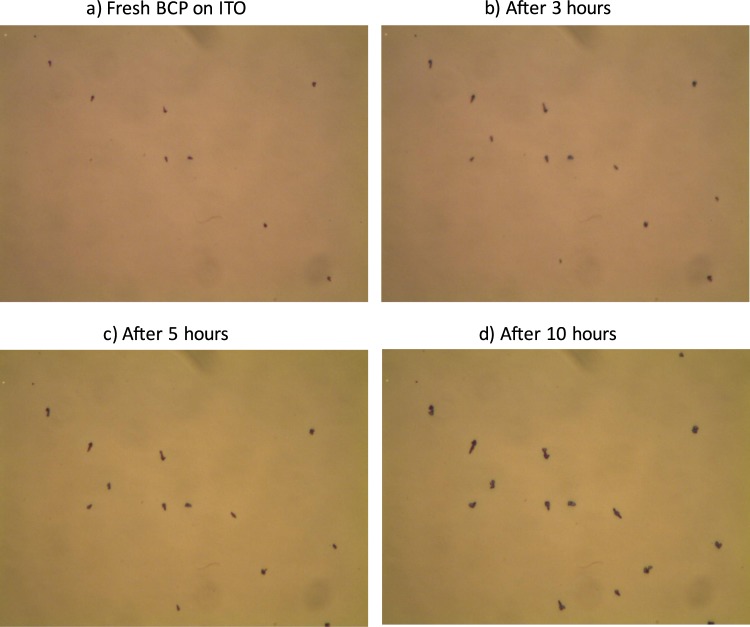
Optical microscope images (100X magnification) recorded at the same spot over time showing ripening of 3 nm BCP clusters on an ITO surface.

In order to investigate the origin of the decreasing device yield and performance in inverted OPV devices, morphological investigations of the BCP layers were carried out. The optical microscope images of the BCP layer on top of the large area ITO substrates (Fig. 5a) feature spots that correspond to BCP aggregates. Such an aggregation of BCP on ITO is due to a large interfacial energy between ITO and BCP^28^, leading to a Volmer-Weber type growth^29^. We suggest that these aggregated BCP structures on ITO are responsible for the shunting of the OPV devices, i.e., for device failure and low device yield.

A possible key to understand the relation between the presence of the BCP clusters and the area-dependent device yield is the time-dependence of the cluster formation. Optical microscopy images of 3 nm BCP on ITO recorded at the same spot over time (shown in Fig. 5) reveal that the BCP clusters on ITO grow further in size over time; this increase in cluster size is consistent with Oswald ripening^30,31^. The size of the BCP clusters depends on the overall available surface area due a combination of two aspects: Firstly, the cluster size depends on the initial amount of molecules deposited on the surface. Larger areas enable the deposition of a larger number of molecules. Secondly, initially formed clusters need to pick up molecules from their surrounding areas in order to grow. Hence, larger areas imply a larger probability for having clusters that are with several hundred nanometers in height tall enough to shunt the cells.

**Figure 6:**
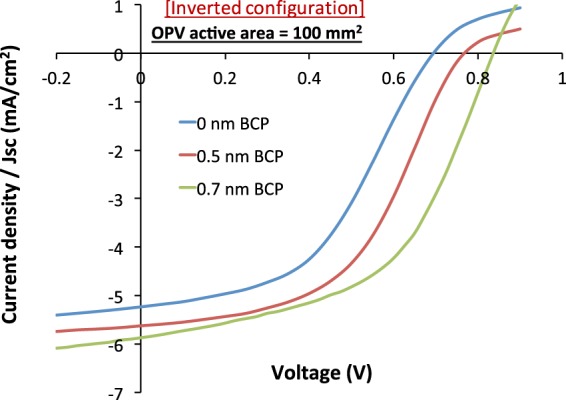
JV characteristics of 100 mm^2^ inverted OPVs with 0, 0.5 and 0.7 nm BCP as ETL.

If clusters tend to be larger for larger electrode areas, then the optimum thickness of BCP also depends on the area of the ITO electrodes. In order to demonstrate this thickness dependence, we fabricated 100 mm^2^ OPV devices in inverted configuration with BCP layer thicknesses reduced further down to 0.7, 0.5, and 0 nm. This reduction improves the overall device yield compared to the 1.5 nm BCP device (cf. Table 5). Their JV characteristics (Fig. 6) and performance values (Table 5) reveal that the device with 0.7 nm BCP (green curve in Fig. 6) has an even larger PCE than the device with 1.5 nm BCP (Table 4). Reducing the BCP thickness beyond 0.7 nm leads to less favorable device performance, which is in line with the behavior previously seen for the 2 mm^2^ area cells.

**Table 5:** The performance parameters of 100 mm^2^ inverted OPVs with BCP ETL of 0, 0.5 and 0.7 nm.

BCP thickness (nm)	Voc (mV)	Jsc (mA/cm^2^)	FF (%)	PCE (%)	Device yield (%)
0	623 ± 48	5.42 ± 0.21	46 ± 06	1.55 ± 0.35	>90%
0.5	761 ± 15	5.52 ± 0.09	49 ± 01	2.06 ± 0.08	≈50%
0.7	826 ± 15	5.83 ± 0.05	51 ± 01	2.46 ± 0.11	<50%

**Figure 7:**
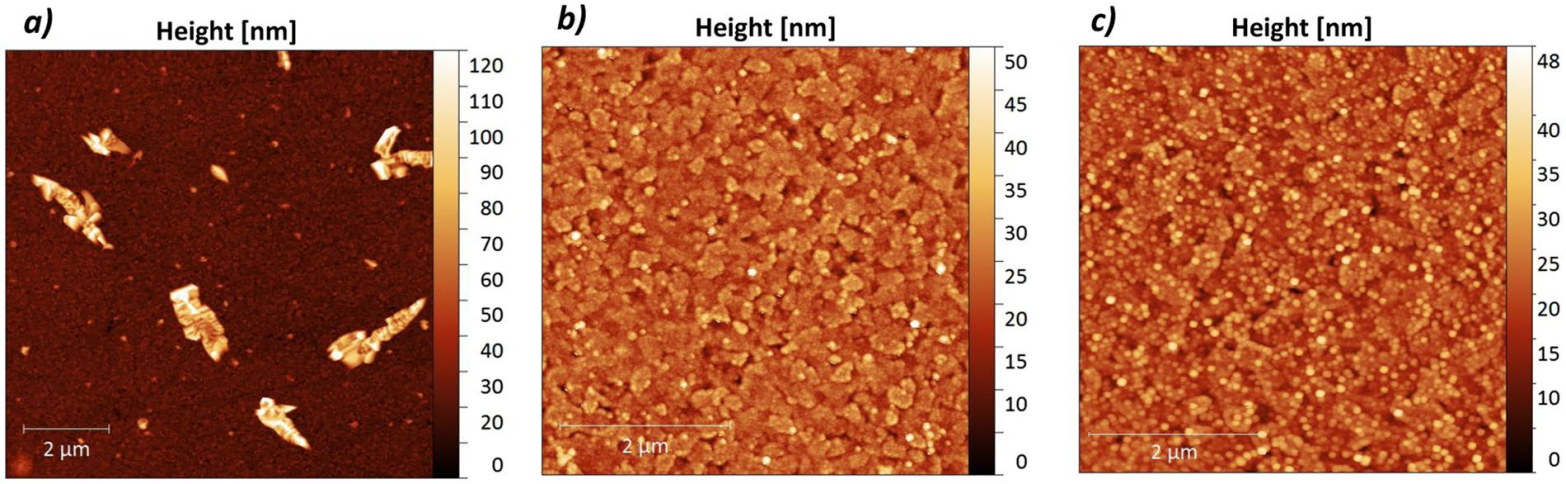
AFM images of (**a**) 3 nm BCP on ITO (inverted configuration); (**b**) 10 nm BCP on 30 nm C_70_, i.e. on top of layer stack of Glass/ITO/MoO_3_(10 nm)/DBP(20 nm)/C_70_(30 nm) (standard configuration) and (**c**) 3 nm BCP on top of layer stack of Glass/ITO/C_70_ (2 nm) (inverted configuration).

The up-scaling of well-performing inverted OPV devices suffers severely from the aggregation of BCP on ITO and thus an increasing probability of device failure. Despite an increased performance, the 100 mm^2^ devices with 0.7 nm BCP thickness were fabricated with a yield of <50% being almost a factor 2 lower than the for the 2 mm^2^ devices. To check to which extent the standard configuration devices may be prone to BCP cluster-induced shunting, we compare Atomic Force Microscopy (AFM) images of 3 nm BCP deposited on ITO (Fig. 7a) and 10 nm BCP deposited on C_70_ (Fig. 7b), corresponding to inverted and standard cell configuration, respectively. While the image in Fig. 7a confirms the formation of BCP clusters on ITO surfaces, no aggregation is discernible on top of C_70_ layers (Fig. 7b). This lack of clusters explains the high device yield for up-scaled standard configuration BCP based cells, and the low device yield for the inverted devices. In the supplementary information provided, AFM scans demonstrate that BCP clusters peaks through the 50 nm thick DBP/C_70_ active layer, which explains the origin of the inverted OPV shunting.

**Figure 8:**
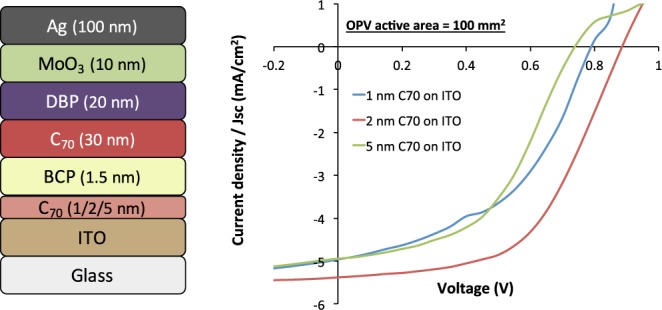
(*Left*) Inverted OPV device stack where ultrathin layers of C_70_ with various thickness are sandwiched between ITO and BCP in order to avoid clustering of BCP and (*Right*) JV characteristics of the fabricated inverted OPV device stack.

Combining the need to suppress the BCP cluster formation and the absence of clusters on C_70_ surfaces readily suggests a route to improve the structure of inverted OPV devices. An ultrathin layer of C_70_ was inserted in between the ITO and the BCP layer, as seen in Fig. 8 (*left*). The performance of the fabricated up-scaled inverted configuration cells is shown in the Fig. 8 (*right*) and Table 6.

**Table 6:** The performance parameters of 100 mm^2^ inverted OPVs where 1, 2 and 5 nm of the C_70_ layer is sandwiched between the ITO and BCP layer.

C_70_ thickness on ITO (nm)	V_OC_ (mV)	J_SC_ (mA/cm^2^)	FF (%)	PCE (%)	Device yield (%)
1	747 ± 63	4.98 ± 0.22	44 ± 05	1.63 ± 0.29	<50%
**2**	**877** ± **10**	**5**.**27** ± **0**.**16**	**54** ± **02**	**2**.**49** ± **0**.**14**	>**70%**
5	777 ± 52	4.74 ± 0.12	45 ± 03	1.66 ± 0.13	>70%

As seen from Table 6, adding 1 nm of C_70_ between the ITO and BCP layers already improves the performance of the inverted cells compared to 100 mm^2^ area devices without BCP (Table 4), both in terms of device performance and yield. Increasing the thickness of the C_70_ layer to 2 nm improves the device yield of the 100 mm^2^ devices further up to 70%, which is significantly higher compared to the 10% device yield of the inverted OPVs without the C_70_ interlayer between ITO and BCP (Table 4). Furthermore, the performance of the inverted devices with 2 nm C_70_ layer sandwiched between ITO and BCP also improves significantly, showing average PCE of 2.49 ± 0.14%, i.e., close to that of a standard configuration device at the same device area. The AFM image of 3 nm BCP on top of a layer stack of glass/ITO/C_70_ (2 nm) is shown in Fig. 7c, revealing no aggregation of BCP here, although smaller BCP peaks are present. The lack of aggregation explains the improved device performance and yield for this structure. Increasing the thickness of the sandwiched C_70_ layer further decreases the overall performance of the OPV devices due to increased series resistance, energy level misalignment, and absorption loss in the active layer, which seems to be minimum at the very low C_70_ layer thicknesses. The results indicate that the proposed method of adding ultrathin films of C_70_ between ITO and BCP layer could be used for large area inverted cells, as it directly addresses the issues related to the aggregation of BCP on ITO, and thus the following device shunting that hampers both performance and especially device yield for large area inverted cells.

## Conclusion

The performance of standard and inverted DBP and C_70_ based OPV devices incorporating BCP as electron transport layer was investigated while up-scaling the device areas from 2 to 10 and 100 mm^2^. The maximum PCE for 2 mm^2^ standard cells was 4.06 ± 0.10%, achieved for BCP thicknesses close to 10 nm, whereas an impressive PCE of 4.48 ± 0.13% for inverted cells was obtained with 1.5 nm BCP, despite the lack of electron-transport mediating metal-complexes inside the BCP layer.

In general, scaling up the device areas led to a drop in the performance that is predominantly caused by an increasing ITO resistance. For standard configuration OPV cells, we found no other factors that affect the device performance; the overall device yield remained high. For the inverted OPV cells, however, the overall device yield was strongly affected by scaling up the cell area. This pronounced dependence on the cell area is ascribed to the formation of BCP clusters on the ITO surfaces upon evaporation. The BCP clusters were observed to grow with time (presumably by Ostwald ripening). The clusters reach heights of up to several hundred nanometers and cause inverted OPV devices to fail due to electrical shunting. The larger the area of the ITO electrodes, the higher the probability that clusters large enough to shunt the cells are formed. Lowering the overall BCP thickness and the amount of BCP molecules evaporated onto the surface improved the device performance. Nevertheless, the yield remained close to 50% for these large area cells, showing that cluster formation still hampers device development.

Ultrathin layers of C_70_ evaporated onto ITO prior to the deposition of the BCP electron transport layers reduced the clustering of BCP on the surface. From the JV characteristics, it was noted that the device performance of 100 mm^2^ cells improves notably by sandwiching 2 nm of C_70_ between ITO and BCP, and also the device yield was observed to be larger than 70%, which was the highest observed for this cell area. Therefore, the presented approach has the potential to improve the device yield and performance to a significant extent for the large area inverted OPV cells incorporating BCP as ETL. In the future, co-evaporation of BCP and C_70_ together as ETL in the fabrication of inverted OPVs could be a promising direction for improving the device yield even further.

## Methods

For 2 and 10 mm^2^ OPVs, 100 nm ITO coated glass wafers (University Wafer, Inc., USA) were patterned by positive photolithography and ITO etching, while for 100 mm^2^ OPV devices, ITO coated glass substrates (Kintec Company, Hong Kong) were obtained pre-patterned. ITO coated glass substrates were cleaned sequentially in an ultrasonic water bath with Acetone and IPA for 10 min each. Substrates were then dried using N_2_ gas.

For the inverted (structure shown in Fig. 1a) 2 mm^2^ OPV devices fabricated on the cleaned ITO substrates, BCP with the thicknesses of X = 0, 1.5, 3 and 5 nm was deposited at a growth rate of 0.2 Å/s. This was followed by 30 nm C_70_ (Sigma-Aldrich, Germany) at a growth rate of 0.5 Å/s and 20 nm DBP (Luminescence Technology Corp., Taiwan) deposited at 0.3 Å/s by Organic Molecular Beam Deposition (OMBD) at a base pressure of 3 × 10^−8^ mbar. MoO_3_ (Sigma-Aldrich, Germany) and Silver (Ag) (AESpump ApS, Denmark) were deposited by thermal evaporation at a base pressure of 5 × 10^−7^ mbar at a rate of at 0.3 Å/s and 1 Å/s, respectively. For the standard (as per the structure shown in Fig. 1b) 2 mm^2^ OPV devices, layers with a sequence of MoO_3_ (10 nm), DBP (20 nm), C_70_ (30 nm), BCP (Y = 0, 3, 5, 10, 15, 20 and 30 nm) and Ag (100 nm) were evaporated by keeping the deposition parameters the same as for the inverted OPV devices. For the up-scaled devices with the cell area of 10 and 100 mm^2^, devices for both standard and inverted configuration were fabricated using the optimized BCP thicknesses for the respective small area cell configurations.

The current density-voltage (JV) characteristics of OPV devices were measured in ambient conditions by applying a voltage sweep from +2 to −1 V using a 2400 source measure unit (Keithley Instruments Inc., USA), and a class AAA solar simulator (Sun 3000, Abet Technologies Inc., USA) having a calibrated lamp intensity of 100 mW/cm^2^. For 2 mm^2^ Electron-Only-Devices (EODs) (structure shown in the inset of Fig. 3), BCP with the thicknesses of X = 0, 1.5, 3 and 5 nm was deposited on the cleaned ITO substrates, followed by 30 nm C_70_ and 100 nm Ag keeping the deposition parameters exactly same as for the OPVs. The JV characteristics of EODs were measured in ambient air by applying a voltage sweep from +1 to −1 V using a 2400 source measure unit (Keithley Instruments Inc., USA).

Optical microscope images were taken using an Industrial microscope eclipse LV100D optical microscope (Nikon Corporation, Japan). AFM images of the structures were scanned using a Veeco Dimension 3100 scanning probe microscope.

## Electronic supplementary material

Supplementary Information
