# Supplementary material for: Area dependent behavior of bathocuproine (BCP) as cathode interfacial layers in organic photovoltaic cells
Source: Sci Rep. 2018 Aug 22;8:12608. doi: 10.1038/s41598-018-30826-7 (PMC6105677; doi:10.1038/s41598-018-30826-7)
Supplement: Supplementary file 1 — Supplementary Information [file 41598_2018_30826_MOESM1_ESM.docx]

**Area dependent behavior of bathocuproine (BCP) as cathode interfacial layers in organic photovoltaic cells**

Bhushan R. Patil^1^, Mehrad Ahmadpour^1^, Golnaz Sherafatipour^1^, Talha Qamar^1^, Antón F. Fernández^1,2^, Karin Zojer^2^, Horst-Günter Rubahn^1^ and Morten Madsen^1^*

^1^SDU nanoSYD, Mads Clausen Institute, University of Southern Denmark, Alsion 2, 6400 Sønderborg, Denmark.

^2^Institute of Solid State Physics and NAWI Graz, Graz University of Technology, Petersgasse 16, 8010 Graz, Austria. *Correspondence and requests for materials should be addressed to M.M. (email: [madsen@mci.sdu.dk](mailto:madsen@mci.sdu.dk))


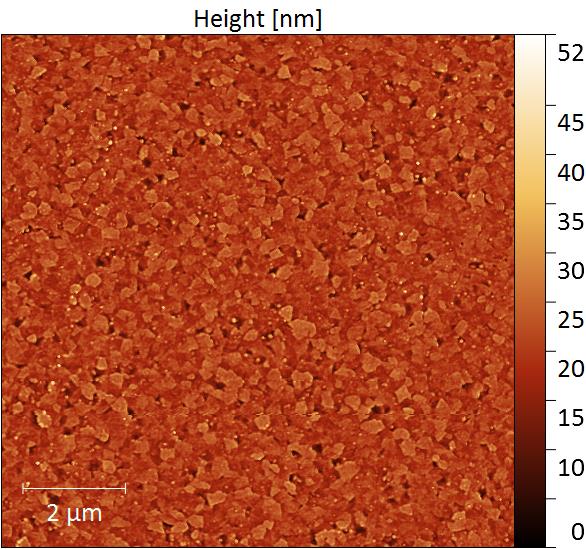


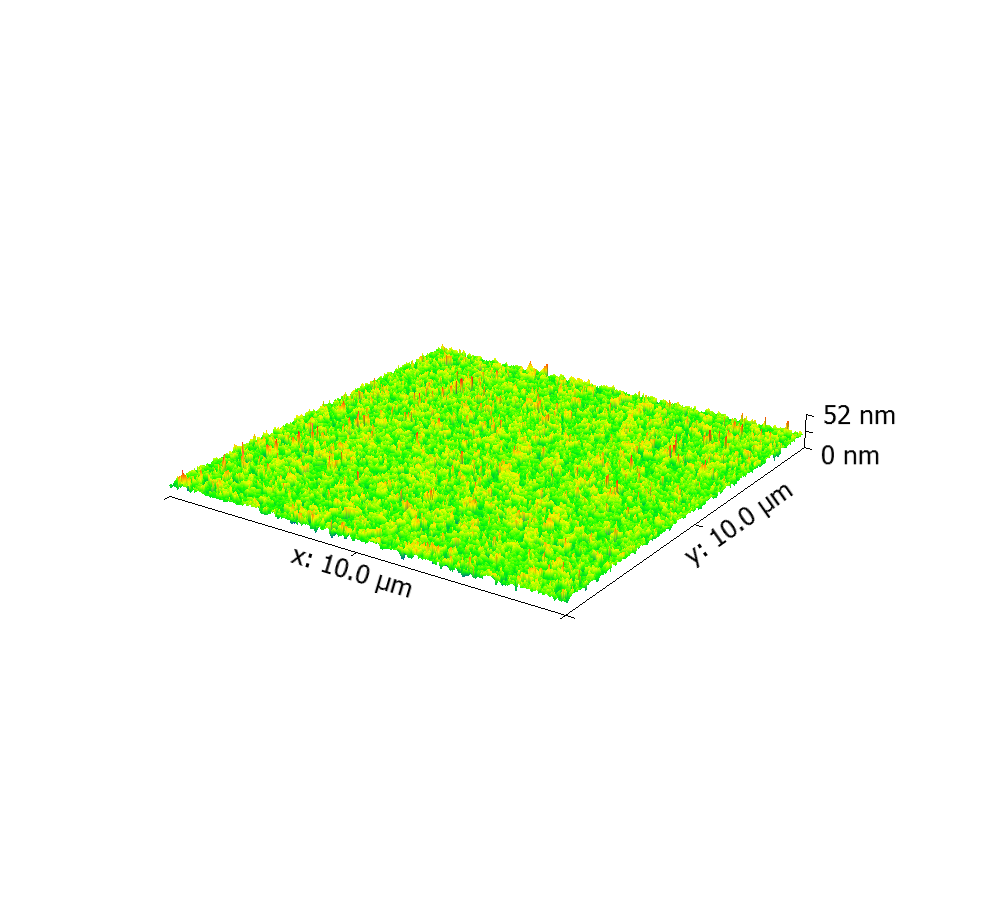


**Figure S1.** *(top)* 2-Dimensional (2D) and *(bottom)* corresponding 3-Dimensional (3D) AFM image of the active layer (DBP/C_70_) morphology on top of 3 nm BCP layer in inverted configuration OPV layer stack, i.e. glass/ITO/BCP(3nm)/C70(30nm)/DBP(20nm).

2D and 3D AFM images in figure S1 show the morphology of bilayer DBP/C_70_ (50 nm) active layers on top of 3 nm BCP coated ITO substrates (inverted configuration). Roughness peaks of up to around 50 nm in height are observed, which are believed to be the high peaks arising from the BCP clusters, as seen in figure 7*a* in the main manuscript. It is observed that the major parts of the individual BCP clusters are covered by the active layer, leaving only the very high but small BCP cluster peaks (higher than total active layer thickness of 50 nm) uncovered, which ultimately results in the electrical shunting of the OPV devices, causing the decreased device yield.
